# Thymoquinone-loaded lipid vesicles: a promising nanomedicine for psoriasis

**DOI:** 10.1186/s12906-019-2675-5

**Published:** 2019-11-27

**Authors:** Poonam Negi, Ishita Sharma, Chetna Hemrajani, Charul Rathore, Alpna Bisht, Kaisar Raza, O. P. Katare

**Affiliations:** 1 School of Pharmaceutical Sciences, Shoolini University of Biotechnology and Management Sciences, Solan, 173 212 India; 2 Department of Pharmacy, School of Chemical Sciences and Pharmacy, Central University of Rajasthan, Bandarsindri, Distt., Ajmer, 305817 India; 3 University Institute of Pharmaceutical Sciences, UGC Centre of Advanced Study, Punjab University, Chandigarh, 160014 India

**Keywords:** Cold method, Ethosomal vesicle, *Nigella sativa*, Ex vivo, Permeation, Mouse tail model

## Abstract

**Background:**

Psoriasis, a recurrent, chronic inflammatory disorder of skin, is a common problem in middle age and elderly people. Thymoquinone (TQ), a lipid soluble benzoquinone is the major active ingredient of volatile oil of *Nigella sativa* (NS), possesses good anti-psoriatic activity. However, its hydrophobicity, poor aqueous solubility, and photosensitive nature obstructs its development. Therefore, in the present research work, ethosomal vesicles (EVs) loaded with TQ were assessed for its anti-psoriatic potential employing mouse-tail model.

**Methods:**

TQ-loaded EVs were prepared by cold method, and characterized for various essential attributes, viz. particle size, morphology, percent drug entrapment, flexibility, rheological and textural analysis, and skin absorption. The optimized formulation was finally evaluated for anti-psoriatic activity on Swiss albino mice employing mouse-tail model for psoriasis.

**Results:**

The spherical shaped vesicles were in the nanosize range, and had high flexibility. The EVs incorporated hydrogel was rheologically acceptable and resulted in substantial TQ retention in the skin layers. The % anti-psoriatic drug activity was observed to be substantially better in the case of TQ-loaded ethosomal gel *vis-à-vis* plain TQ, NS extract, and marketed formulation.

**Conclusions:**

The promising outcomes of the current studies ratify the superiority of TQ-loaded phospholipid-based vesicular systems for the management of psoriasis over other studied test formulations. This study, thus open promising avenues for topical application of TQ in the form of EV hydrogel.

## Background

Psoriasis, a skin disorder characterized by unraced cutaneous, demolition, agitated proliferation and poor isolation of epidermal keratinocytes. It is a recurrent, chronic inflammatory problem, and is associated with severe morbidity and social stigma making it an impending threat [1–3]. The prevalance of psoriasis worldwide ranges between 0 and 6.6%, whereas in India, it has been recently reported as high as 8% [4, 5]. A broad range of treatment options involving systemic therapy [6], topical therapy [7]*,* and phototherapy [8]*,* are available and practised. Amid these, the topical therapy is generally preferred owing to the ease of application, localized impact and reduced systemic load [9], esp. in the cases when the body surface area covered is not more than 50%*.*

*Nigella sativa* (NS) which is an annual flowering plant, belonging to family Ranunculaceae, has been used for centuries for the treatment of many skin disorders including psoriasis. Much of the anti-psoriatic activity of NS seeds has been attributed to the presence of thymoquinone (TQ), which is one of the major component of essential oil but also present in the fixed oil. Topical administration of TQ, which is a lipid soluble benzoquinone, has attracted special attention, as it exerts the therapeutic action against several skin pathologies, viz. acne vulgaris, skin pigmentation, vitiligo, hypersensitivity reactions, early stages of skin tumorigenesis including psoriasis [10]. TQ exerts its effect by acting as a free radical and superoxide radical scavenger, meanwhile preserving the activity of various anti-oxidant enzymes such as catalase, glutathione peroxidase and glutathione-S-transferase [11]. Despite of having promising benefits for skin therapeutics, complete clinical utilization of TQ has been limited owing to its high hydrophobicity, poor aqueous solubility, poor bioavailability, chemical instability, and its poor penetration through the system [12]. Therefore, an effective delivery is needed to increase the solubility, protect the drug and at the same time facilitate penetration and retention in the hyperkeratotic skin of psoriatic lesions.

Currently, researchers are being focused towards the development of newer delivery approaches in order to get enhanced benefit using TQ for the treatment of psoriasis. Jain et al., 2017 have encapsulated TQ in the lipospheres and evaluated the anti-inflammatory and anti-psoriatic potential using in vitro cell lines and imiquod induced plaque psoriasis model. Results indicated the improvement in the phenotypic, histopathological features and reduced levels of IL-17 and TNF-α in psoriatic skin [13]. Ali et al., 2019 formulated TQ lipid nanoparticles and optimized employing Box-Behnken design. Psoriasis area and severity index score confirmed the reduction in all the psoriasis related parameters in imiquod–induced plaque psoriasis model [12].

Vesicular systems could be potential delivery carriers for improving solubility and enhancing therapeutic concentration of drug at the target site [14]. Among different vesicular systems, a novel vesicular system composed of phospholipids, ethanol and water could be effective in fulfilling the therapeutic requirements of TQ [15]. Ethosomes can not only improve the solubility of TQ which will help attain superior drug loading but the highly deformable character of ethosomes could assist penetration in deeper skin layer surmounting the stratum corneum (SC) barrier better than the traditional liposomes. Ethosomes have been reported to improve the drug entrapment, skin penetration and deposition of various drugs. Further, the stability and rheolgical applicability of ethosomes can be enhanced via incorporating in the hydrogel system [16].

Herein, we have designed TQ-loaded ethosomal hydrogel systems and investigated its antipsoriatic potential in mouse-tail model. The permeation and skin absorption potential of the developed system was evaluated through the Wistar Albino rat skin. The rheological and textural attributes of the prepared ethosomal hydrogel was also assesed.

## Methods

### Materials

NS seeds were collected from M/s Balmukand, Solan, Himachal Pradesh. TQ was purchased from M/s Sigma Aldrich, Mumbai, India. Phospholipon 90G was procured *ex gratis* from GmbH, Nattermannallee, Germany. Carbopol 934 was purchased from M/s Lubrizol, Mumbai, India, while ethanol from Changshu Yangyuan Chemical, China. All Other ingredients and reagents were of analytical grade.

The study was carried out as per CPCSEA (Committee for the purpose of control and supervision of experiment on animals) guidelines. The experimental protocol was duly approved by Institutional Animal Ethical Committee of Shoolini University (IAEC/SU-PHARM/16/03). The swiss albino mice (20–25 g) used in the psoriasis model, Wistar Albino rats (150–250 g) used in skin permeation studies were, procured from Animal house facility of National Institute of Pharmaceutical Education and Research, Mohali, Punjab, India. Then the animals were kept at the animal house establishment of Shoolini University, Solan, HP, India which is in validation with ARRIVE guidelines prescribed for in vivo experimentation on small animals. The animals were maintained at the temperature of 25 ± 2 °C, with relative humidity of 45 ± 5%, and provided with water and food ad libitum. The animals were initially acclimatized to experimental laboratory conditions for 7 days before testing.

#### Preparation of NS extract

NS seeds were purchased from Solan, Himachal Pradesh, India and authenticated from Department of Forestry, YS Parmar University of Horticulture and Agriculture Sciences, Nauni, HP, India. The voucher specimen no. is SU-634. The seeds were thoroughly cleaned with water to remove dust particles and dried in shade. The dried seeds were first powdered and, then used for extraction by boiling 200 g of seed powder in 1000 mL of 95% ethanol, for 2 days, in a soxhlet extraction unit. The extract obtained was vaporized and concentrated on a water bath at atmospheric pressure to a semisolid condition. The semisolid concentrate was taken in a shallow dish and was kept in an oven at 30 °C for further drying to completely remove the solvent. The NS extract so obtained was used in anti-psoriatic activity on mouse tail model.

#### Preparation of TQ-loaded EVs

TQ-loaded EVs (TQ EV 1- TQ EV 6) (Table 1) were formulated by well-reported cold method [16]. In brief, the Phospholipon 90G (3–4%) along with TQ (200 mg) was dissolved in ethanol (10–30%) to yield a transparent solution. This ethanolic solution was added in a smooth fashion to distilled water (q.s to 100%) and was constantly stirred at 900 rpm (M/s REMI Equipments, Mumbai, India). Stirring was further continued for 5 mins to form whitish EV dispersion. The suspension was kept at room temperature for 2 h for absolute hydration (i.e., swelling of phospholipids), prior to conducting any characterization and evaluation studies on it [17].

**Table 1:** Composition and various evaluation parameters of ethosomal formulations

Formulation Code	Phospholipid^a^ (mg)	Ethanol %	Particle size (nm)	% PDE^b^	% PDL^c^	%Transmittance	Sedimentation volume
TQ EV 1	300	10	253.7	45.00 ± 0.75	30.00	64.0	0.4
TQ EV 2	300	20	308.0	62.00 ± 0.53	41.00	71.5	1
TQ EV 3	300	30	395.4	59.88 ± 0.64	37.92	60.0	0.8
TQ EV 4	400	10	477.6	79.52 ± 0.24	39.76	73.5	0.1
TQ EV 5	400	20	507.7	70.96 ± 0.19	35.48	65.0	0.3
TQ EV 6	400	30	531.3	62.28 ± 0.53	31.14	57.0	0.5

^a^*Phospholipid* : Phospholipon G, ^b^*PDE* : Percent Drug Entrapment, ^c^*PDL* : Percent Drug Loading

#### Micromeritics

Particle size characterization of all the prepared EVs was carried out on Malvern’s Zetasizer (Nano ZS, U.K) installed at University Institute of Pharmaceutical Sciences, Panjab University, Chandigarh, India. The instrument contains a 4 mV He-Ne laser source working at a wavelength of 633 nm and incorporating non-invasive black scatter optics (NIBS). The measurements were performed at detection angle of 173°, and the measurement site inside the cuvette was automatically determined by the software. A 5 mL of the sample was placed in the cuvette and instrument recorded the intensity of fluctuation of laser beam, which was automatically interrelated to the particle size of vesicle [18].

#### Transmittance studies

Transmittance of different EV formulations was observed spectrophotometrically at a wavelength of 254 nm by diluting 0.2 mL of the suspension upto 10 mL with water against water as the blank (set at 100% transmittance) [19].

#### Microscopic characterization

To study the morphology of vesicles and appearance of any aggregates, agglomerations or unentrapped particulate drug, the EVs were examined under the compound microscope (Radical, RXL-5, B-8692, India).

#### Transmission electron microscopy

Morphology and structure of the EVs were also determined with the help of Transmission Electron Microscope (TEM) installed at Central Instrumentation Lab Panjab University, Chandigarh. Following the negative staining with a 1% aqueous solution of phosphotungstic acid (PTA), vesicular systems were dried on a microscopic carbon-coated grid and viewed under the microscope at a suitable magnification. Microphotographs of the EVs were clicked at appropriate magnification [18].

#### Flexibility index

Membrane flexibility index (FI) of EV carriers was determined using vesicle-extruder (Eastern Sci. Inc., MD, USA). Concisely, the vesicular suspension was passed through polycarbonate membrane of 0.05 μ pore size using vesicle extruder. The vesicle size and size distribution measurement of EVs was observed before and after extrusion, employing Delsa Nano particle size analyzer (M/s. Beckman Coulter Inc., CA, USA). FI was then calculated using Eq. (1).

1 $$ FI=\frac{PE- PA}{PA} $$

Where PE and PA are the values of particle size before and after extrusion, respectively [18].

#### Percent drug entrapment (PDE) and percent drug loading (PDL)

The PDE of TQ-loaded EVs was also determined by ultracentrifugation method Remi CPR 24 Plus, India. Vesicular preparations were centrifuged at 23,980 g for 2 h, at 4 °C. Clear supernatant as well as the vesicular sediment was assayed for the drug (TQ) content. PDE of TQ was calculated using Eq. (2).

2 $$ PDE=\frac{\left(T-C\right)}{T}\times 100 $$

Where, T is the total amount of drug that is detected both in the supernatant and sediment, and C is the amount of drug detected only in the supernatant [20]. PDL was calculated with respect to loading of the drug per total solid mass with weight of the drug and lipid taken initially.

3 $$ PDL=\frac{Amount\ of\ Drug\ in\  EV}{\  Total\ mass\ of\ Drug\ and\ Lipid}\times 100 $$

#### Sedimentation volume

The EVs suspension prepared were kept in 10 mL measuring cylinder, after 24 h, the volume of sedimentation was observed [21].

#### Development of secondary topical vehicle

The optimized EV suspension was further incorporated in Carbopol 934 hydrogel (1% w/w). Carbopol as a gelling agent might cause rise in residence time of a drug at a target site of absorption by interacting through the skin mucosa [22]*.*

#### Rheology and texture analysis

The viscosity of the prepared EV gel was determined by means of a cup and bob rheometer at 25 °C (Anton Paar Rheometer HS 143; M/s Anton Paar, Ashland, VA). Briefly, sample was placed in the concentric cylindrical assembly and shear stress (Ƭ) value was constantly increased linearly from 0.1 to 100 s^− 1^. The data analysis was performed by Herschel-Bulkey models Eqs. (4) and (5) [23].

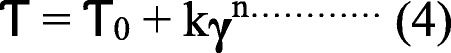

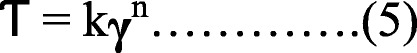





Where, k = consistency index (Pa sec^n^); Ƭ_0_ = yield stress (Pa); n = power-law exponent. TQ-loaded ethosomal gel formulations were also evaluated for texture properties with the use of Texture Analyzer™ (TA. XT. Plus Texture Analyzer; M/s Stable Microsystems, Surrey, UK). Accurately weighed (5–6 g) sample was placed in the lower cone. The upper cone probe was calibrated against the lower cone prior to testing. Throughout the testing, upper cone probe penetrated into the sample and inter leaved to a depth of 2 mm with test speed of 3 mm/s [19, 24]. A variety of textural parameters was then intended from the variation of force as a function of time.

### Ex vivo skin permeation studies

The ex vivo drug permeation studies was performed on excised dorsal skin of Albino rat of Wistar albino strain (150–250 g) on a two-compartment diffusion cell assembly made in-house (cross-sectional area of 3.142 cm^2^). Abdominal rat skin tissue was mounted, with the stratum corneum surface facing upwards, and the donor compartment was set aside open to atmosphere. The skin tissue was permissible to equilibrate with the sink medium (30 mL of Phosphate buffer saline 7.4 containing 30% v/v Ethanol). All the formulations (1 g) viz., TQ suspension in normal saline pH 7.4 and TQ-loaded EV, each containing TQ equivalent to 20 mg (i.e. 2% w/w) were applied evenly, on the dorsal side of skin, in donor compartment. Aliquots of 1 mL were withdrawn periodically for a period of 5 h, and replaced with the same volume of the receptor medium, to continue the receptor phase volume at a steady level. At the end of this study (5 h), the donor compartment and the skin surface(s), were washed thrice with receptor medium. Samples obtained were properly diluted, and quantified for drug using UV-spectrophotometric analysis. Drug permeation behavior, of the test formulations was studied for various skin transport characteristics, viz. permeation flux (J), and percent cumulative drug permeation (% CDR) [25].

#### Drug retention studies

After accomplishment of the permeation studies, the skin samples fixed on diffusion cells were removed carefully, and washed thrice, to remove any adhered formulation. The skin was then cut into small pieces, mixed with 5 mL ethanol and shaken for 5 h at 30 ± 1 °C for complete extraction of TQ. Supernatant was filtered through a membrane (0.22 μm). Subsequently, the filtrate was analyzed by UV-spectrophotometer at λ_max_ of 253 nm for drug concentration after suitable dilutions.

#### Anti-psoriatic activity in mouse-tail

Mouse-tail skin is unusual model as it undergoes both parakeratotic and orthokeratotic keratinization in adjacent sites. The former develops without a granular layer and resembles psoriasis, while the latter, with a granular layer, resembles normal human skin. Based on this property, mouse-tail skin has frequently been used as a model for psoriasis [26]. Healthy albino mice, aged between four to 6 weeks and weight between 20 and 25 g were used to determine anti-psoriatic activity employing mouse-tail model. Animals were randomly divided into five groups, each containing three mice (*n* = 3), given normal saline (group 1), plain TQ suspension (group 2) (20 mg/kg), NS extract (group 3) (20 mg/kg), TQ-loaded EV gel (group 4) (20 mg/kg) and marketed anti-psoriatic cream, i.e., Tazarotene© (group 5) (0.05%w/w) respectively. The formulations were applied once daily to proximal half of the mouse-tail for 2 weeks. Mice were sacrificed by spinal dislocation after 24 h of last application of formulation(s), and the tail skin was removed by longitudinal dissection with a scalpel. The obtained skin samples were duly processed, stained with hematoxylin–eosin and were examined under microscope for the presence of granular layer in the scale regions and epidermal thickness. Orthokeratosis induced in sections of the adult mouse-tail, having normal parakeratotic differentiation was evaluated. Five sequential scales were examined for each skin section. Percent orthokeratosis was calculated using Eq. (6).

6 $$ \% Orthokeratosis=\frac{\mathrm{A}}{\mathrm{B}}\times 100 $$

Where A = length of the granular layer, B = length of the scale.

Drug activity (DA) was calculated using Eq. (7):

7 $$ DA=\frac{Mean\  OK\  of\ treated\ group- Mean\  OK\  of\ control\ group\ X100}{100- mean\  OK\  of\ control\ group} $$

Where OK represents percent orthokeratosis [24].

## Results

### Characterization of TQ-loaded EVs

**Fig. 1:**
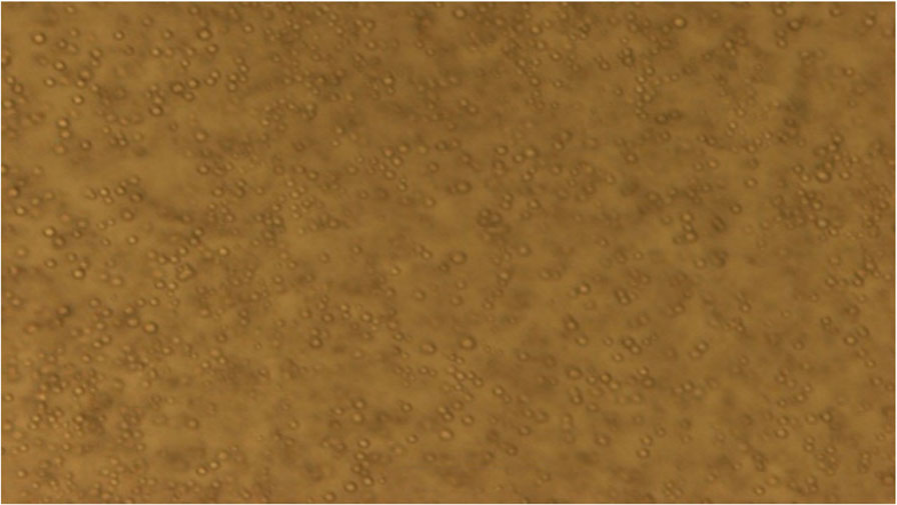
Microscopic evaluation of ethosomal dispersion (1000X)

**Fig. 2:**
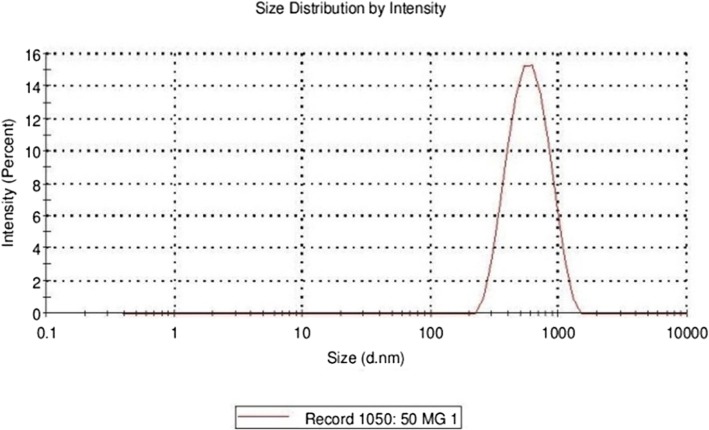
Particle Size Distribution for Formulation EV 4

**Fig. 3:**
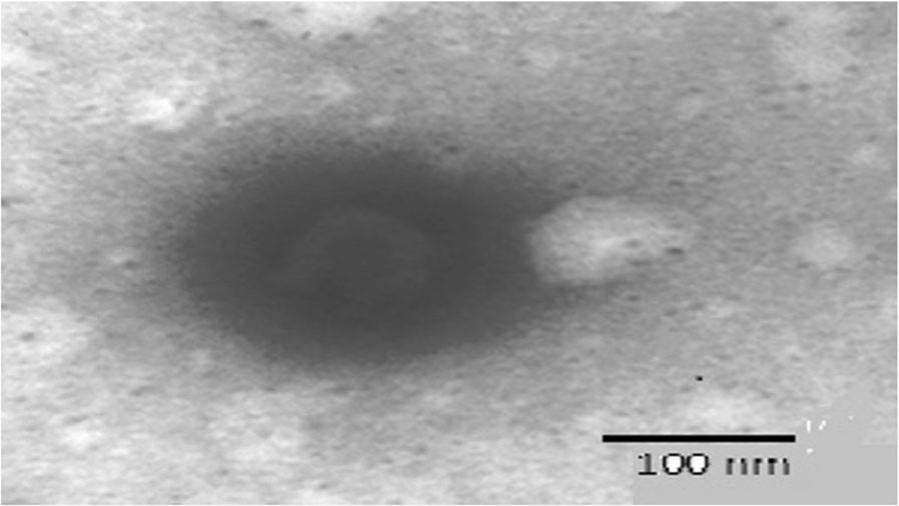
TEM micrograph (21,000 X) of EV4 formulation

The prepared EVs were studied for optical microscopy and the optical microscopic image of ethosomal vesicle EV4 is depicted in Fig. 1. The vesicles were present in much abundance, having spherical morphology, size uniformity with less aggregation and fusion. The vesicle size of EVs was found to be in the nanometer range of 253.7 nm to 531.3 nm, as depicted in Table 1.The vesicle size distribution of EV4 is also shown in Fig. 2, indicating uniformity of size. PDE was found to be in the range of 45–62% and PDL was found in the range of 30–41%. It was observed that the amount of phospholipid and ethanol significantly affected both vesicle size as well as PDE. In the case of vesicle size, increase in the amount of phospholipid and ethanol significantly enhanced the size, while increase in ethanol concentration from 10 to 20% at both the phospholipid levels, increased the PDE followed by a slight decrease at 30% ethanol concentration. The % transmittance values for all the prepared EVs were observed below 74 indicating possibility of high vesicle density. Among the different formulations prepared, the ethosomal suspension, i.e., TQ EV 4 has revealed the lowest sedimentation volume and thus was more stable in comparison to all the other formulations as shown in Table 1. The TEM microscopic image of the EV4 formulation (Fig. 3) clearly depicts that the vesicles were spherical and in nanometric range. FI was determined employing the vesicle extruder (Eastern Sci. Inc., MD, USA) to assess the membrane flexibility of the EVs and is an essential and specific parameter of EVs. It differentiates EVs from other conventional vesicular carriers like liposomes in terms of degree of flexibility and hence, ability to cross the stratum corneum. The EVs demonstrated the FI value of 3.29, which is very high and indicates the high flexibility of the vesicles.

#### Rheology and texture analysis

The values of various rheological parameters obtained for the EV hydrogel is shown in Table 2 i.e. viscosity (η), flow consistency index (K), flow behavior index (n), yield value, gel strength, spreading coefficient, stickiness. As the value of “n”was found to be less than 1 (i.e., 0.254), the developed hydrogel system was declared as shear-thinning in nature. Consistency index for EV gel was observed to be 42.055 Pa with apparent viscosity of 31.07 Pa.s. The yield value was found to be on the higher side, i.e., 73.61 Pa, which may be ascribed to the rigidity of hydrogel structure of the EV gel, thus requiring higher shear to initiate its flow. Further, the values of various obtained parameters reveal that the EV gel exhibits fairly good gel strength (17.55 Kg), ease of spreading (6.1 Kg.s), lesser stickiness (1.16 kg.sec). The textural results of EV hydrogel meet the desired characteristics for any topical formulation.

**Table 2:** Rheological characteristics of the developed Ethosomal Gel formulation

Formulation Parameter	Ethosomal Gel
^a^n	0.254
^a^K (Pa)	42.055
^a^η (Pa.sec)	31.07
Yield value (Pa)	73.61
Gel strength (Kg)	17.55
Work of spreading (Kg.sec)	6.1
Stickiness (Kg.sec)	1.16

^a^n: Flow Behavior Index; K: Flow Consistency Index; η = Viscosity

#### Ex vivo skin permeation and retention studies

The permeation behavior of TQEV4 formulation through the skin was compared with that from TQ suspension. The % cumulative skin drug permeation and various other skin transport characteristics like flux, permeability coefficient values of TQEV4 and TQ suspensions are portrayed in Table 3. All the transport values for TQEV4 were on the higher side vis-à-vis TQ suspension. The % cumulative skin drug permeation was 36.6% for TQEV4 and 11.6% for TQ suspension. The flux of TQ for TQEV4 was 11.2 μg /cm^− 2^ h^− 1^ in comparison to TQ suspension for which the value was 1.0 μg/cm^− 2^ h^− 1^. Similarly permeability coefficient values for TQEV4 was also higher (15.7 × 10^− 3^) in comparison to TQ suspension (8× 10^− 3^). Further, the amount retained in the skin from TQ EV (88.71 (μg/cm^2^) was also higher in comparison to TQ suspension (72.86 (μg/cm^2^).

**Table 3:** Skin-transport parameter obtained for various test formulations (Mean ± SD)

Formulation	Flux (μg /cm^− 2^ h^− 1^)	PC_0_ × 10^–3 a^	Cumulative drug skin-permeation (%)	Amount retained in skin (μg/cm^2^)
TQ EV	11.25 ± 1.05	15.78 ± 2.01	36.62 ± 2.01	88.71 ± 3.02
TQ suspension	1.08 ± 0.01	8.00 ± 1.01	11.60 ± 1.07	72.86 ± 3.01

^a^ *PC*_*0*_: Permeability coefficient

The superior permeation and retention profile from EVs could be ascribed to the carrier effect, i.e., better entrapment efficiency of TQ in ethosomes, enhanced fluidity possessed by these carriers and a synergistic mechanism between ethanol, phospholipid vesicles and skin lipids leading to an enhanced permeation and retention.

### Anti-psoriatic activity in mouse-tail

**Fig. 4:**
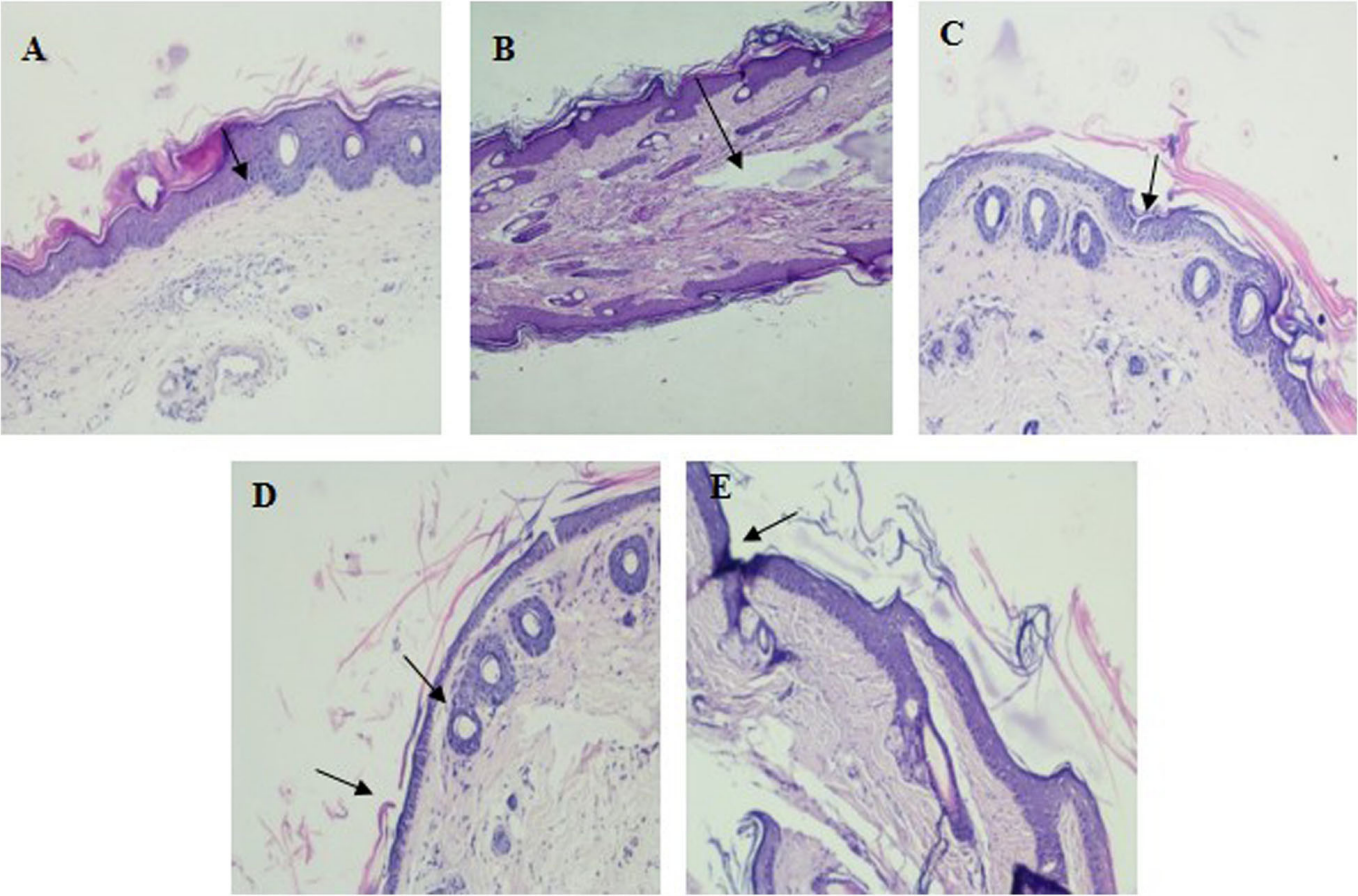
Histology of the orthokeratotic mouse tail skin action: parakeratotic skin section of the mouse tail at 40x(**a**) Saline (**b**) Thymoquinone (**c**) *Nigella sativa* Extract (**d**) TQ loaded EV gel E) Tazarotene™

Histological changes in epidermal keratinization are presented in Fig. 4. Further % orthokeratosis and % DA values for all the treatments are shown in Table 4. Saline-treated skin (control), Fig. 4a reveals almost all the characteristic features of the parakeratotic skin with lamellar horny layer having high density of nucleated cells and presence of no granular layer. Treatment with a various formulation for 2 weeks had shown marked histological changes in the mouse-tail skin, as the regions of orthokeratotic SC (i.e. with blue staining) extended longitudinally into the regions of previously parakeratotic SC. The granular layer was clearly visible and compact horny layer revealed presence of normal population of nucleated cells. % OK and % DA values of TQ and TQ-loaded EVs gel revealed much better anti-psoriatic activity. It was clearly visible from the photograph (Fig. 4d) that there was less parakeratotic layer left in case of EV gel treated tail skin as compared to the pure TQ (Fig. 4b), NS extract (Fig. 4c), and marketed formulation (Fig. 4e). It was also implied that there was greater conversion of the parakeratotic skin layer to the orthokeratotic layer on the application of TQ-loaded EV gel as compared to the marketed formulation, plain TQ or NS extract [27].

**Table 4:** % Drug activity of different formulations (Mean ± SD)

Group no.	Formulation	% Orthokeratosis	% Drug activity
1	Normal saline	44 ± 1.05%	0
2	TQ suspension	51.73.2 ± 2.15%	17.1 ± 1.03%
3	*Nigella sativa* extract	50.56 ± 3.01%	13 ± 1.25%
4	TQ loaded EV Gel	55.23 ± 2.05%	25 ± 2.35%
5	Tazarotene™	51.17 ± 2.03%	19.2 ± 1.05%

% Orthokeratosis, which is an indicator of the efficacy of anti-psoriatic effect, was in the order TQ loaded EV gel (55.23 ± 2.05%) > TQ Suspension (51.73.2 ± 2.15%) > Tazarotene (51.17 ± 2.03%) > *Nigella sativa* Extract (50.56 ± 3.01%).

**Fig. 5:**
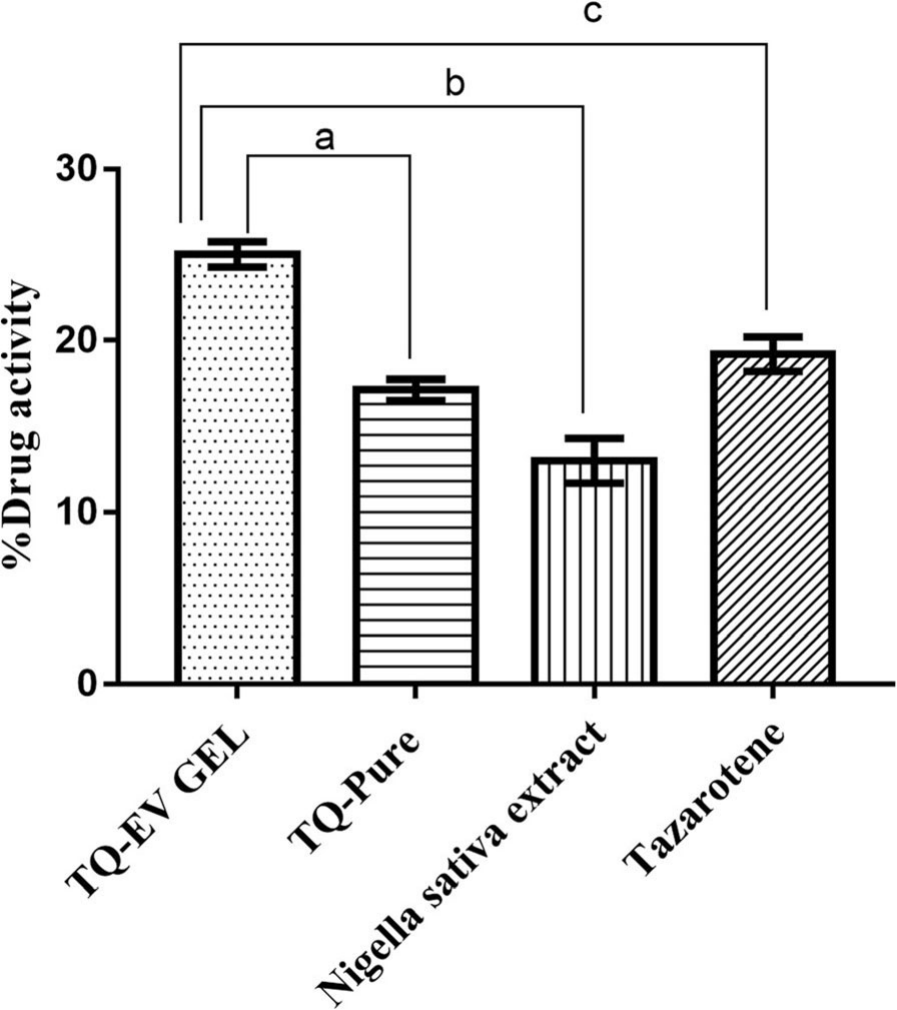
Effect of TQ-loaded EV gel, TQ suspension, *Nigella sativa* and Tazarotene on % Drug activity. Results were shown as mean ± standard error mean analyzed via. One way ANOVA followed by Dunnett’s multiple comparison tests. ^*a*^*P* < 0.001 vs TQ suspension; ^*b*^*P* < 0.001 vs *Nigella sativa* extract and; ^*c*^*P* < 0.001 vs Tazarotene

Similarly % DA (Fig. 5), which directly demonstrates the anti-psoriatic efficacy, was in the order TQ loaded EV gel (25 ± 2.35%) > Tazarotene (19.2 ± 1.05%) > TQ Suspension (17.1 ± 1.03%) > *Nigella sativa* Extract (13 ± 1.25%).

Thus histological evidences, % OK and % DA values indicate the superior efficacy of TQ loaded EV gel over TQ suspension, *Nigella sativa* extract, and Marketed formulation i.e. Tazarotene.

## Discussion

The phospholipid based vesicular systems having ethanol in the bilayer offer deeper skin penetration, while offering high drug loading for hydrophobic drug. The incorporation of TQ into EVs can enhance the therapeutic concentration of the drug at the affected site, prolong the release and facilitate deeper skin penetration.

EVs were prepared by cold method. To select the best EV formulation, different EV formulations were prepared varying phospholipid and ethanol concentrations. The formulations were then studied for vesicular size, % PDE, %PDL, % Transmittance, and Sedimentation Volume. Vesicle size plays a key role in the overall performance of the vesicular carriers [28]. The smaller vesicular size (253–531 nm) of the prepared EVs could be attributed to the presence of ethanol, which results in solubilization of the phospholipids [29]. However, an increase in average vesicular size with increased phospholipid can be ascribed to the formation of large multilamellar vesicles at the concentrations far above the critical micelle concentration of the phospholipids [30, 31]. With all ethanol concentrations, increase of PDE was observed as phospholipid amount increased. This might be related to the enhanced entrapment of the lipophilic drug. However the enhanced entrapment was seen upto 20% ethanol concentration, but then again there was a decrease [32]. The plausible reason for the decrease is more solubility offered to the drug by the hydration medium, which resulted in slight extraction of drug from the interiors of vesicles. Out of all the EV formulations, TQEV4 having high entrapment efficiency (79.52%), optimum particle size (477.6 nm) and low sedimentation volume (0.1 ml) was selected. TQEV4 was found to have spherical morphology with high flexibility. The high value for flexibility index could be explained owing to the significant presence of ethanol in the bilayers of ethosomes, which increase the softness and malleability, vis-à-vis conventional liposomes. Ethosome contain 10–40% of ethanol, which is an optimum concentration to enhance the flexibility of lipidic bilayers and thus increase the dermal penetration. Our selected formulation contain only 10% ethanol, which is safe for the topical administration [25, 33].

The various rheological parameters suggested the prepared TQEV hydrogel to have shear-thinning nature. The yield value was found to be on the higher side (73.61 Pa), indicating the rigidity of the hydrogel structure, thus requiring higher shear to initiate its flow. The textural attributes were found to be of desired characteristics and thus developed EV hydrogel is optimum for topical administration.

As seen from the results of ex vivo permeation studies it was observed that the drug transport characteristics of TQ EV were superior to that of TQ aqueous suspension. The cause for that being the ability of EVs to control the drug transfer across the skin. Adsorption and fusion of EVs on the skin surface, enhances the thermodynamic activity gradient of the drug at the interface, which acts as the driving force for the permeation of a lipophilic drug [33]. The reduction of the barrier properties of stratum corneum results from the property of vesicles as a penetration enhancer [29]. Furthermore, the anti-psoriatic efficacy of TQ was confirmed by histopathological evaluation of mouse-tail. Histological examination of mouse skin lesion revealed parakeratosis and orthokeratosis, which resembled the skin lesion of human psoriatic skin [34]. Results showed that the EV gel formulation were able to enhance the anti-psoriatic efficacy of TQ and its percent orthokeratosis induction was comparable to that of the marketed product. However, %DA of TQ-loaded EV gel was better than all the other formulations including the marketed one (Fig. 5). Nano size range of EVs owing to their higher surface area maintain a closer contact with the epithelium to be able to sustain the appreciable amount of drug during the entire period of treatment in animals.

## Conclusions

The promising outcomes of the current studies ratify the superiority of TQ-loaded novel phospholipid-based systems in the treatment of psoriasis over the conventional gel. The vesicular carriers proved to be soft, flexible and site targeted vesicles and elicited the enhanced penetration and retention of TQ, in comparison to the conventional systems. The % drug activity was revealed better in the case of TQ loaded ethosomal gel in comparison to the NS extract, TQ alone and tazarotene gel. The results obtained in the present studies, thus open newer avenues for topical application of TQ in the treatment of psoriasis.

## Data Availability

All data generated or analyzed during this study are included in this published article.

## Abbreviations

%CDR: Percent Cumulative Drug Permeation
CPCSEA: Committee for the purpose of control and supervision of experiment on animals
DA: Drug Activity
EVs: Ethosomal Vesicles
FI: Flexibility Index
HaCaT: Human Keratinocytes Cell Line
NIBS: Non-Invasive Black Scatter Optics
NS: *Nigella sativa*
OK: Orthokeratosis
PDE: Percent Drug Entrapment
PDL: Percent Drug Loading
PTA: Phosphotungstic Acid
SC: Subcutaneous
TEM: Transmission Electron Microscopy
TQ: Thymoquinone
